# Cross-Sectional Study on Prevalence and Molecular Characteristics of Plasmid Mediated ESBL/AmpC-Producing *Escherichia coli* Isolated from Veal Calves at Slaughter

**DOI:** 10.1371/journal.pone.0065681

**Published:** 2013-05-28

**Authors:** Joost Hordijk, Jaap A. Wagenaar, Arie Kant, Alieda van Essen-Zandbergen, Cindy Dierikx, Kees Veldman, Ben Wit, Dik Mevius

**Affiliations:** 1 Department of Infectious Diseases and Immunology, Faculty of Veterinary Medicine, Utrecht University, Utrecht, The Netherlands; 2 Central Veterinary Institute, part of Wageningen UR, Lelystad, The Netherlands; 3 Netherlands Food and Consumer Product Safety Authority, Utrecht, The Netherlands; Iowa State University, United States of America

## Abstract

**Objectives:**

The presence of ESBL/AmpC-producing *E. coli* in cattle has been reported previously, however information on veal calves is limited. This study describes the prevalence and molecular characteristics of *E. coli* with non-wild type susceptibility to cefotaxime in veal calves at slaughter.

**Methods:**

Faecal samples from 100 herds, 10 individual animals per herd, were screened for *E. coli* with non-wild type susceptibility for cefotaxime. Molecular characterization of ESBL/AmpC genes and plasmids was performed on one isolate per herd by microarray, PCR and sequence analysis.

**Results:**

66% of the herds were positive for *E. coli* with non-wild type susceptibility for cefotaxime. Within-herd prevalence varied from zero to 90%. 83% of *E. coli* producing ESBL/AmpC carried *bla*_CTX-M_ genes, of which *bla*_CTX-M-1_, *bla*_CTX-M-14_ and *bla*_CTX-M-15_ were most prevalent. The dominant plasmids were IncI1 and IncF-type plasmids.

**Conclusions:**

A relatively high prevalence of various *bla*_CTX-M_ producing *E. coli* was found in veal calves at slaughter. The genes were mainly located on IncI1 and IncF plasmids.

## Introduction

The development of resistance to extended spectrum cephalosporins (ESC) has evolved rapidly world-wide in both clinical settings as well as in the community. Resistance to ESC is mainly caused by various extended spectrum ß-lactamases (ESBL) or AmpC ß-lactamases (AmpC) that are often found in *Enterobacteriaceae* and hydrolyse the ß-lactam ring of these antibiotics [1]. These enzymes are encoded by genes that are frequently located on mobile genetic elements (plasmids) [2], which have the ability to transfer horizontally within and between different bacterial species. Novel ESBL/AmpC genes or gene variants are reported on a regular basis (http://www.lahey.org/studies) and are classified based on either their functional characteristics [3] or primary structure [4].

Food-producing animals have been suggested as the primary reservoir of zoonotic foodborne pathogens, including antimicrobial resistant bacteria [5]. Faecal carriage of ESBL/AmpC-producing *E. coli* in cattle has been reported previously [6], [7], [8], [9], [10]. In addition, a recent study showed that in the period from 2005 to 2010, resistance to ESC in veal calves was mainly caused by plasmid mediated beta-lactamases, in contrast to the years before 2005 (Hordijk *et al*, submitted for publication). Furthermore, beef, chicken and pig meat products have also been found to be positive for ESBL/AmpC-producing bacteria [11], which may constitute a transmission route to humans. However, studies are difficult to compare, because isolation methods and sample sizes vary greatly. When cattle is studied, the type of cattle included is not always specified. For instance dairy farming is very different from veal calves by means of housing, life expectation and exposure to antibiotics. The majority of veal farms maintain an all-in, all-out system, while dairy farms generally maintain a closed production system. Almost each individual animal on a veal calf farm originates from another dairy farm. Furthermore, compared to dairy cattle, veal calves are more frequently exposed to antimicrobials [12]. These factors may influence the prevalence and genetic characteristics of antimicrobial resistance genes in isolates from these animals. Furthermore, antimicrobial susceptibility data from national surveillance studies have shown that isolates from veal calves are often multi resistant [12].

Therefore, the aim of this study was to determine the prevalence of *E. coli* with reduced susceptibility to ESC in veal calf herds. Moreover the ESBL/AmpC genes and plasmids on which they are located were identified to determine their molecular characteristics in relation to those found in other food-producing animals and humans.

## Materials and Methods

### Sampling design and isolation of Escherichia coli

From January to December 2011, fecal samples from 10 individual veal calves were taken from 100 slaughter batches (1000 samples in total). The samples were taken as part of the Dutch national control program on antimicrobial resistance by the Netherlands Food and Consumer Product Safety Authority (NVWA). All slaughter batches originated from different herds. To ensure anonymity, the origin of samples was aggregated at province level. The sampled slaughter batches were equally distributed over the five slaughter houses in the Netherlands and the sampling took place equally distributed over the year. All fecal samples were individually screened for ESC reduced susceptible *E. coli* using selective enrichment broth. Fecal swabs were taken from the colon of the carcass after evisceration and subsequently transferred to the laboratory and placed in a tube with 1 ml Luria-Bertani broth (Beckton Dickinson) supplemented with 1 mg/L cefotaxime (Sigma-Aldrich, Germany) (LB+). LB+ tubes were incubated aerobically at 37°C for 20±2 hours. Subsequently, the overnight LB+ culture was inoculated on a MacConkey agar plate (Becton Dickinson), which was supplemented with 1 mg/L cefotaxime (MC+). All MC+ plates were incubated aerobically at 37°C for 20±2 hours. From each MC+ plate showing growth, one colony typical for *E. coli* was selected, subcultured on blood agar, and suspended the next day in 1 ml of buffered peptone water supplemented with 30% glycerol and stored at −80°C, pending analysis. All selected *E. coli* were inoculated in 5 ml Gersbach medium and incubated at 37°C for 20±2 hours for *E. coli* confirmation. One droplet of Kovac's Indole reagent (Merck, Germany) was added to the Gersbach culture to check for tryptophan reduction following the requirements for *E. coli* identification stated by the European Food Safety Authority (EFSA) [13]. All indole-positive isolates were considered *E. coli*. From all isolates showing growth on MacConkey, one randomly picked isolate was selected per herd for antimicrobial susceptibility testing and molecular characterization.

### Antimicrobial susceptibility testing

All selected isolates (n = 66) were tested for susceptibility to antimicrobials by broth micro-dilution according to ISO standard 20776-1:2006 using microtitre trays with a custom made dehydrated panel of antibiotics (Sensititre, Trek Diagnostic Systems, Basingstoke, UK). The following antibiotics were included: ampicillin, cefotaxime, ceftazidime, tetracycline, sulfamethoxazole, trimethoprim, ciprofloxacin, nalidixic acid, chloramphenicol, florfenicol, gentamicin, kanamycin, streptomycin and colistin. All results were interpreted using cut-off values defined for epidemiological purposes as recommended by the European Committee on Antimicrobial Susceptibility Testing (EUCAST; http://mic.eucast.org/Eucast2/), apart from sulfamethoxazole, for which the clinical break point defined by the Clinical and Laboratory Standards Institute (CLSI) was used [14]. Values above these cut-offs and break points were interpreted as resistant (sulfamethoxazole) or non-wild type (all other antimicrobials) [15].

### Characterization of ESBL/AmpC genes and plasmids

All selected isolates were screened for ESBL/AmpC genes using the tube based amr-ve-05 microarray (Alere, Tilburg, the Netherlands) [16]. Beta-lactamase gene families identified by this microarray were subsequently characterized by PCR and sequence analysis as described previously [17]. For sequence analysis the following additional primers were used: TEM-Fseq: 5′-GCCAACTTACTTCTGACAACG, CMY-F-838: 5′-TGGCGTATTGGCGATATGTA and CMY-R-857: 5′-TACATATCGCCAATACGCCA. Primers and conditions used to identify and sequence the modified aminoglycoside resistance gene *aac(6′)Ib-cr* have been described previously [18]. Plasmids were isolated using a modified miniprep method as follows. One colony of interest was inoculated in 3 ml LB broth and incubated overnight. Subsequently 1.5 ml of the culture was transferred and spinned down at 14000 rpm for 5 minutes. The pellet was suspended in 60 µl TEG buffer (25 mM Tris/Hcl; 10 mM EDTA; 50 mM glucose). Then, 120 µl NaOH/SDS (0.2 M/1%) was added and the whole sample was placed on ice for 5 minutes. Subsequently, 90 µl NaOH (3 M) was added and the sample was placed on ice for 5 minutes. The suspension was spinned down at 14000 rpm for 5 minutes. The supernatant was transferred and 270 µl LiCl (5 M) was added. After 10 minutes, the suspension was spinned down at 14000 rpm for 5 minutes. The supernatant was transferred and 1 ml of EtOH (96%) was added. The suspension was spinned down at 14000 rpm for 10 minutes and the pellet was resuspended in 200 µl EtOH (70%). The suspension was spinned down at 13000 rpm for 10 minutes and the pellet was resuspended in 20 µl H_2_O. Plasmids were transformed into Electromax DH10B cells by electroporation (Invitrogen, USA) by mixing 2 µl of plasmid DNA suspension with 20 µl competent cells. The cells were electroporated under the following conditions: 1.25 kV, 200 ohm, 25 µFar. Transformants were subsequently plated on LB agar plates supplemented with 1 mg/L cefotaxime in order to selectively isolate transformants with ESBLs carrying plasmids. PCR-based replicon typing (PBRT) was conducted on the transformants to identify the replicon type of the plasmid inside the transformant [19]. Plasmid MLST (pMLST) [20] and replicon sequence typing (RST) were used to further characterize IncI1 and IncF plasmids, respectively [21]. Plasmids that were negative in the PBRT analysis were designated “not typable” (nt). The size of these plasmids was determined by PFGE with S1-nuclease digestion as described previously [22]. Because this study focused on plasmid mediated genes, another ESBL/AmpC suspected isolate from the same herd was included as a replacement if the micro array results were negative. For isolates in which no plasmid mediated ESBL/AmpC gene was detected, the promoter region of the chromosomal *ampC* gene was sequenced using primers that have been described previously [23]. Specific *ampC*-types were designated as described by Mulvey *et al* [24]. In addition a combination disk test was performed as described previously on all isolates that were negative in the array [17].

### Ethics Statement

Samples were taken from the colon of the veal calves after slaughter as part of the Dutch national control program on antimicrobial resistance as required by the zoonosis directive of the European Parliament (2003/99/EC). Since the sampling of calves was not performed on live animals, no approval from the ethical committee was required. In line with ‘Regulation (EC) No 854/2004 of the European Parliament and of the Council, article 4 section 8’, concerning specific rules for the organisation of official controls on products of animal origin intended for human consumption, the NVWA is the designated authority in the Netherlands to take samples for surveillance purposes. The NVWA granted permission to analyse the samples as described in this paper.

## Results

### Prevalence of non-wild type cefotaxime susceptible E. coli

From the 100 herds that were screened for the presence of *E. coli* with non-wild type susceptibility at slaughter, 66% were found positive for *E. coli* with non-wild type susceptibility to cefotaxime. The within-herd prevalence of *E. coli* with non-wild type susceptibility varied greatly, ranging from zero to 90%. However, in the majority of the positive farms, less than five out of ten fecal samples harbored *E. coli* with non-wild type cefotaxime susceptibility. From all positive farms 90% was located in the eastern and southern provinces. This is also where veal farming is concentrated. In addition, 86% of the ESBL/AmpC-producing *E. coli* was multi-drug resistant, showing non-wild type susceptibility to three or more antimicrobial classes, including ß-lactams (Tables 1 and 2).

**Table 1:** *E. coli* carrying *bla*_CTX-M_ group 1 genes with their corresponding plasmids and resistance profile.

			Plasmid		
ESBL	Strain	Other ß-lactamase	Replicon	incI1 pMLST/incF RST 1, 2	Non-wild type susceptibility^3^
CTX-M-01	OT-ESBL-0199	TEM-1a, OXA-1	inc nt	(97 kb)^5^	Amp-Ctx-Caz-Tet-Chl-Str-Kan
	OT-ESBL-0294		inc nt	(97 kb)^5^	Amp-Ctx-Caz-Smx-Tmp-Gen
	OT-ESBL-0285		incB/O		Amp-Ctx-Caz-Tet-Smx-Tmp-Str-Kan
	OT-ESBL-0547		incB/O		Amp-Ctx-Caz-Tet-Smx-Gen-Str-Kan
	OT-ESBL-0589		incB/O		Amp-Ctx-Caz-Tet-Smx-Tmp-Cip-Nal-Chl-Str
	OT-ESBL-0054		incF	F2; A-; B-	Amp-Ctx-Caz-Tet-Smx-Ffn-Chl-Gen-Str
	OT-ESBL-0198		incF	F2; A-; B-	Amp-Ctx-Caz-Tet- Gen-Str
	OT-ESBL-0591		incF	F2; A-; B-	Amp-Ctx-Caz-Tet-Ffn-Chl-Str
	OT-ESBL-0600		incF	F17*; A-; B-	Amp-Ctx-Caz-Tet-Gen
	OT-ESBL-0382		incF	F13#; A-; B20#	Amp-Ctx-Caz-Tet-Smx-Str-Kan
	OT-ESBL-0519		incF	F35#; A-; B-	Amp-Ctx-Caz-Tet
	OT-ESBL-0565		incF	F35#; A-; B_	Amp-Ctx-Caz-Tet
	OT-ESBL-0328		incI1	ST3; CC 3	Amp-Ctx-Caz-Smx-tmp
	OT-ESBL-0359		incI1	ST3; CC 3	Amp-Ctx-Caz-Tet-Smx-Tmp-Str
	OT-ESBL-0406		incI1	ST58; CC 58	Amp-Ctx-Caz-Tet-Smx-Str-Kan
	OT-ESBL-0018		incI1	ST58; CC 58	Amp-Ctx-Caz-Tet-Smx-Tmp-Str
	OT-ESBL-0062		incI1	new; 1 2 8# 3 3	Amp-Ctx-Caz-Tet-Cip-Nal-Chl-Str
	OT-ESBL-0441		incI1	new; 1 5* 17 1 7	Amp-Ctx-Caz-Tet-Str
	OT-ESBL-0450		incI1	new; 1 5?16 1 7	Amp-Ctx-Caz-Tet-Smx-Cip-Nal-Chl-Str
	OT-ESBL-0477		incI1	new; 1 9# 17 1 7	Amp-Ctx-Caz
	OT-ESBL-0546		incK		Amp-Ctx-Caz-Tet-Smx-Cip-Nal-Chl-Str-Col
	OT-ESBL-0262	OXA-1	incN		Amp-Ctx-Caz-Tet-Smx-Tmp-cip-Nal-Ffn-Chl-Str-Kan
	OT-ESBL-0361	TEM-1b	incN		Amp-Ctx-Caz-Tet-Smx-Ffn-Chl-Str-Kan
	OT-ESBL-0434		incN		Amp-Ctx-Caz
CTX-M-01	OT-ESBL-0414		incI1	ST58; CC 58	Amp-Ctx-Caz-Tet-Smx-Tmp-Str-Kan
+ TEM-52c			unknown	(145 kb)^5^	
CTX-M-03	OT-ESBL-0437	TEM-1b	incB/O		Amp-Ctx-Caz-Tet-Smx-Tmp-Cip-Nal-Gen-Str-Kan
	OT-ESBL-0567		incF	F35#; A-; B-	Amp-Ctx-Caz-Tet-Smx-Tmp-Cip-Nal-Str-Kan
CTX-M-15	OT-ESBL-0327		inc nt	(110 kb)^5^	Amp-Ctx-Caz
	OT-ESBL-0502	qnrS^4^ (other plasmid)	inc nt	(40 kb)^5^	Amp-Ctx-Caz-Cip
	OT-ESBL-0028		incF	F2; A-; B-	Amp-Ctx-Caz-Tet-Smx-Tmp-Cip-Nal-Str
	OT-ESBL-0031	OXA-1, (aac6′-Ib-cr)^4^	incF	F31; A4; B1	Amp-Ctx-Caz-Tet-Cip-Nal-Kan
	OT-ESBL-0156	OXA-1, (aac6′-Ib-cr)^4^	incF	F31; A4; B1	Amp-Ctx-Caz-Tet-Smx-Tmp-Cip-Nal-Gen-Str-Kan
	OT-ESBL-0221	OXA-1, (aac6′-Ib-cr)^4^	incF	F31; A4; B1	Amp-Ctx-Caz-Tet-Smx-Tmp-Cip-Nal-Gen-Str-Kan
	OT-ESBL-0161	TEM-1b, OXA-1, (aac6′-Ib-cr)^4^	incF	F31; A4; B1	Amp-Ctx-Caz-Tet-Smx-Tmp-Cip-Nal-Gen-Str-Kan
	OT-ESBL-0563	OXA-1, (aac6′-Ib-cr)^4^	incF	F31; A-; B1	Amp-Ctx-Caz-Tet-Smx-Tmp-cip-Nal-Ffn-Chl-Gen-Str-Kan
	OT-ESBL-0534	TEM-1b	IncF	F46; A-; B20	Amp-Ctx-Caz-Tet-Smx-Tmp-cip-Nal-Ffn-Chl-Gen-Str-Col
	OT-ESBL 0256		IncI1	ST31; CC 31	Amp-Ctx-Caz-Tet-Smx-Tmp-cip-Nal-Ffn-Chl-Gen-Str-Kan
	OT-ESBL-0549		incI1	ST31; CC 31	Amp-Ctx-Caz-Cip-Nal
	OT-ESBL-0443		incHI2		Amp-Ctx-Caz-Tet-Smx-Tmp-Cip-Nal-Ffn-Chl-Gen-Kan
CTX-M-32	OT-ESBL-0163		inc nt	(40 kb)^5^	Amp-Ctx-Caz-Tet-Smx-Tmp-Cip-Nal-Chl-Str-Kan
	OT-ESBL-0173		inc nt	(40 kb)^5^	Amp-Ctx-Caz-Tet-Smx-Tmp-Cip-Nal-Chl-Str-Kan
	OT-ESBL-0449		inc nt	(40 kb)^5^	Amp-Ctx-Caz-Tet-Smx-Tmp-Cip-Nal-Ffn-Chl-Str-Kan
	OT-ESBL-0473		inc nt	(40 kb)^5^	Amp-Ctx-Caz-Tet-Smx-Tmp-Cip-Nal-Chl-Str-Kan

1 The following deviations were found in IncI1 pMLST types: ESBL-0062: trbA8#: nucleotide 371 T>C; ESBL-0441: ardA5*: 59 G>A, 137 A>T, 271 C>G, 331 C>T; ESBL-0450: ardA5?: 199 G>A, 271 C>G, 331 C>T; ESBL-0477: ardA9#: 199 G>A/C

2 The following deviations were found in IncF RST types: ESBL-0301: FIB-26#: 251 G>T; ESBL-0382: FII-13#: 77 A>G and FIB-20#: 194 G>A, 203 T>C; ESBL-0519/0565/0567: FII-35#: 22 A>T, 38+39>CG insertion.

3 Amp  =  ampicillin, Ctx  =  cefotaxime, Caz  =  ceftazidime, Nal  =  nalidixic acid, Cip  =  ciprofloxacin, Ffn  =  florfenicol, Chl  =  chloramphenicol, Tet  =  tetracycline, Smx  =  sulfamethoxazole, Tmp  =  trimethoprim, Str  =  streptomycin, Kan  =  kanamycin, Gen  =  gentamicin), Col  =  colistin.

4 *qnrS* and *aac*(*6*′)*Ib-cr* are not ß-lactamase genes, but cause reduced susceptibility to quinolones. *aac*(*6*′)*Ib-cr* also causes reduced susceptibility to aminoglycosides.

5 Plasmid size in kilo bases (kb) of non-typeble plasmids.

**Table 2:** *E. coli* carrying ESBL/AmpC genes other than *bla*_CTX-M_ group 1 with their corresponding plasmids and resistance profile.

			Plasmid		
ESBL	Strain	Other ß-lactamase	Replicon	incI1 pMLST/incF RST	Non-wild type susceptibility^1^
CTX-M-02	OT-ESBL-0301	TEM-1b	incF	F1; A6; B26#	Amp-Ctx-Caz-Tet-Smx-Tmp-cip-Nal-Ffn-Chl-Gen-Str-Kan
	OT-ESBL-0310		incHI1		Amp-Ctx-Caz-Tet-Smx-Tmp-Ffn-Chl-Gen-Kan
	OT-ESBL-0514		incP, incHI2		Amp-Ctx-Caz-Tet-Smx-Tmp-Ffn-Chl-Str-Kan
CTX-M-14	OT-ESBL-0403		incF	F2; A-; B-	Amp-Ctx-Caz-Tet-Smx-Tmp-cip-Nal-Ffn-Chl-Gen-Str-Kan-Col
	OT-ESBL-0021	TEM-1b	incI1	ST80	Amp-Ctx-Caz-Tet-Smx-Tmp-Cip-Nal-Ffn-Chl-Str
	OT-ESBL-0336		incI1	ST80	Amp-Ctx-Caz-Tet-Smx-Cip-Nal-Ffn-Chl-Str-Kan
	OT-ESBL-0337		incI1	ST80	Amp-Ctx-Caz-Tet-Smx-Cip-Nal-Ffn-Chl-Str-Kan
	OT-ESBL-0058		incK		Amp-Ctx-Caz-Tet-Smx-tmp-Str
	OT-ESBL-0291		incK		Amp-Ctx-Caz-Tet-Smx-Tmp-Str
	OT-ESBL-0380	TEM-1b	incK		Amp-Ctx-Caz-Tet-Smx-Tmp-cip-Nal-Ffn-Chl-Gen-Str-Kan
	OT-ESBL-0405		incK		Amp-Ctx-Caz-Tet-Gen
	OT-ESBL-0590		incK		Amp-Ctx-Caz-Tet-Smx-Tmp-Cip-Nal-Chl-Gen-Str
TEM-52c	OT-ESBL-0192		incI1	ST36; CC 5	Amp-Ctx-Caz
	OT-ESBL-0364		incI1	ST36; CC 5	Amp-Ctx-Caz-Smx-tmp-Cip-Nal-Chl-Str
	OT-ESBL-0392	TEM-1b	incI1	ST10; CC 5	Amp-Ctx-Caz-Tet-Smx-Tmp-Chl-Str
CMY-2	OT-ESBL-0357		incK		Amp-Ctx-Caz-Tet-Str
ampC-type-3	OT-ESBL-0281		-		Amp-Ctx-Caz-Tet-Smx-Str-Kan
	OT-ESBL-0386	TEM-1b	-		Amp-Ctx-Caz-Tet-Smx-Ffn-Chl-Str
	OT-ESBL-0453		*-*		Amp-Ctx-Caz
	OT-ESBL-0599	TEM-1a	*-*		Amp-Ctx-Caz-Tet-Smx-Tmp-Str-Kan
	OT-ESBL-0601	TEM-1b	*-*		Amp-Ctx-Caz-Tet-Smx-Ffn-Chl-Str
*ampC*-type-11var	OT-ESBL-0543	TEM-1a	*-*		Amp-Ctx-Caz-Tet-Smx-Tmp-Str
Unknown	OT-ESBL-0261	TEM-1c, *OXA*-1, ampc WT	-		Amp-Ctx-Caz-Tet-Smx-Tmp-cip-Nal-Ffn-Chl-Str-Col

1 Amp  =  ampicillin, Ctx  =  cefotaxime, Caz  =  ceftazidime, Nal  =  nalidixic acid, Cip  =  ciprofloxacin, Ffn  =  florfenicol, Chl  =  chloramphenicol, Tet  =  tetracycline, Smx  =  sulfamethoxazole, Tmp  =  trimethoprim, Str  =  streptomycin, Kan  =  kanamycin, Gen  =  gentamicin), Col  =  colistin.

Of all selected isolates with a non-wild type susceptibility to cefotaxime, 83% carried genes belonging to the *bla*_CTX-M_ gene family (Table 1+2). The predominant genes within this *bla*_CTX-M_ group, were *bla*_CTX-M-1_ (45.5%), *bla*_CTX-M-14_ (16.4%), and *bla*_CTX-M-15_ (21.8%). One of the isolates harbouring *bla*_CTX-M-1_ also harboured *bla*_TEM-52c_. The remaining *bla*_CTX-M_ variants were *bla*_CTX-M-2/97_ (5.5%), *bla*_CTX-M-3_ (3.6%) and *bla*_CTX-M-32_ (7.2%). From 11 isolates (17%) that did not harbour *bla*_CTX-M_, three harboured *bla*_TEM-52c_ (4.5%), and one *bla*_CMY-2_ (1.5%). In 6 isolates (9%), only mutations in the promoter region of the chromosomal *ampC* were detected. Finally, in one isolate (1.5%) neither plasmid mediated ESBL/AmpC genes nor mutations in the promoter region of the chromosomal *ampC* were detected. This isolate only harboured the ß-lactamases *bla*_TEM-1c_ and *bla*_OXA-1_.

The predominant plasmid types among all isolates harbouring ESBL/AmpC genes were IncI1 and IncF. Both IncI1 and IncF plasmids were present in 26% of the isolates depicted in Tables 1 and 2. The highest diversity of plasmids was observed among *bla*_CTX-M-1_ positive isolates, which was also the most abundant ESBL variant. In 9 isolates, *bla*_CTX-M-1_ was located on IncI1 with six different IncI1 pMLST types (Table 1). From these six different pMLST types, four types had not yet been reported in the pMLST database (http://www.pubmlst.org/plasmid, last accessed: 20 March 2013). Furthermore, in 7 isolates *bla*_CTX-M-1_ was located on IncF plasmids with four different IncF RST profiles. From these four different profiles, two had not yet been reported in the pMLST database. The remaining *bla*_CTX-M-1_ genes were located on IncB/O (three isolates), IncN (three isolates) and IncK (one isolate). In two isolates the plasmid was not typable. The second largest group of ESBL genes, *bla*_CTX-M-15_, was predominantly carried on IncF plasmids, of which four different RST types were found among seven isolates (Table 1). From these seven isolates, five also harboured *bla*_OXA-1_ and *aac*(*6*′)*Ib-cr*. The remaining *bla*_CTX-M-15_ genes were located on IncI1 (two isolates), IncHI2 (one isolate) and two on not typable plasmids. The *bla*_CTX-M-14_ genes were mainly carried on IncK plasmids (Table 2), but also on IncI1, of which all three plasmids had the same pMLST sequence type (ST80), and one on IncF (F2; A-; B-)). From the isolates harbouring *bla*_CTX-M-2/97_ or *bla*_CTX-M-3_, all genes were located on different plasmids (Table 1+2). All plasmids carrying *bla*_CTX-M-32_, were not typable. Furthermore, four isolates harboured *bla*_TEM-52c_, of which three were carried on IncI1 plasmids with two different pMLST types (ST10 and ST36), both belonging to the same clonal complex (CC5) (Table 2). From the fourth *bla*_TEM-52c_, which was found in an isolate also harbouring *bla*_CTX-M-1_, transformation of the plasmid failed, so no replicon type was established. In the single isolate harbouring an *ampC* gene, *bla*_CMY-2_ was located on an IncK plasmid (Table 2). Finally, one transformant harbouring *bla*_CTX-M-2_ showed two replicon types, namely IncP and IncHI2 (Table 2).

## Discussion

This study showed that 66% of the slaughter batches of veal calves were positive for fecal carriage *E. coli* with a non-wild type susceptibility to cefotaxime. In 68% of the positive herds, five or less animals out of ten were found carrier of these isolates. Since only one isolate per herd was included in the molecular analysis, no conclusions can be drawn towards clonal diversity of ESBL/AmpC genes and plasmids in herds with multiple positive animals. Further in depth studies should be performed to assess whether a clonal spread within herds or a high diversity of ESBL/AmpC genes and/or plasmids exists within these herds. Factors that may influence the prevalence and spread of these resistance determinants within herds, including the use of antimicrobials and farm management, should be taken into account to analyze possible differences between farms.

Studies have been performed on fecal carriage of ESBL/AmpC-producing *E. coli* in cattle at slaughter in several countries, and the prevalence varied greatly. In Poland no ESBL/AmpC-producing *E. coli* were observed [25], in contrast to France (5.8%) [10], Switzerland (16%) [26], Hong Kong (3.1%) [27] and Japan (31.3%) [28]. However, while all studies were performed on individual animals, comparing these prevalence data should be performed with care since not all studies used the same selection methods (*e.g*. the use of enrichment broth and/or different selective media), which may have resulted in different screening sensitivities.

The vast majority of plasmid mediated ESBL/AmpC genes in the present study belonged to the *bla*_CTX-M_ gene family, of which *bla*_CTX-M-1_ was most abundant, followed by *bla*_CTX-M-15_ and *bla*_CTX-M-14_ (Table 1+2). These findings are in line with the observed trend in the last five years of a retrospective study performed on faecal samples from veal calves collected at farms from 1997 to 2010 [29]. Studies from other countries, that focused on ESBLs in general or *bla*_CTX-M_ genes in specific, confirm the relatively high abundance of *bla*_CTX-M-1_, *bla*_CTX-M-14_ and/or *bla*_CTX-M-15_ genes in either healthy or sick cattle [6], [8], [10], [30]. In contrast, in the United States, *bla*_CMY-2_ is the predominant gene found in cattle and *bla*_TEM_ ESBL genes were found at a low level [31], [32]. Furthermore, a study from Hong Kong reported that *bla*_CTX-M-13_ was the only ESBL observed in cattle at slaughter [27] and *bla*_CTX-M-2_ was reported to be predominant in cattle in Japan [33]. This indicates that there are geographical differences in the prevalence of resistance genes.

This study showed that ESBL/AmpC genes found in veal calves were predominantly located on IncI1 and IncF plasmids. These plasmids belong to the most commonly reported plasmid families in *Enterobacteriaceae* [34]. Interestingly, a relatively high proportion of IncI1 and IncF plasmid subtypes (mainly associated with *bla*_CTX-M-1_) had not yet reported in the pMLST database (Table 1). IncI1 sequence types (ST) in combination with *bla*_CTX-M-1_ have been reported either commonly (CC3, ST7) or occasionally (ST58) [34], [35], [36]. To our knowledge, the combination of *bla*_CTX-M-1_ with IncF type plasmids has not been reported previously. The IncF RST-type found in this study (F2;A-;B-) has frequently been associated with *bla*_CTX-M-15_ [34], [37]. Furthermore, we have also found *bla*_CTX-M-1_ in combination with IncB/O plasmids. This has also been reported in isolates from humans [38] and horses [11]. The *bla*_CTX-M-1_ associated with IncN was previously observed in human isolates [38], [39], poultry [40], and in both porcine isolates and their farm workers, indicating transmission may occur between food-producing animals and their care takers [41].

In this study, *bla*_CTX-M-15_ was mainly located on IncF plasmids, a combination that has been reported frequently [34], [39], [42], [43]. In addition, the combination of *bla*_CTX-M-15_ with *bla*_OXA-1_ and *aac6*′*-Ib-cr* (the latter causing reduced susceptibility to aminoglycosides and fluoroquinolones), has been reported previously [44]. This combination was observed in the predominant IncF type found in this study carrying *bla*_CTX-M-15_ (F31;A4;B1). Furthermore, both the IncF RST types F2;A-B- and F31;A4;B1 and the IncI1 pMLST type ST31 that carry *bla*_CTX-M-15_ that were reported in this study have also been isolated from cattle in France [37]. The *bla*_CTX-M-14_ genes in our study were mainly located on IncK plasmids. This has also been reported in calves in the UK [45], as well as in isolates from humans and turkey [46], [47]. None of the plasmids harbouring *bla*_CTX-M-32_ were typable by PBRT, making it difficult to draw any conclusions towards genetic characteristics. For these isolates, we determined the size of the ESBL harbouring plasmid. The combination of *bla*_TEM-52_ with IncI1 (ST36) has been reported in cattle in France [48], but both ST36 and ST10 in combination with *bla*_TEM-52_ have also been reported in humans and poultry isolates [38] and are the most commonly found types [34]. This shows that various combinations of ESBL/AmpC genes and plasmids are widely distributed in isolates of both animal and human origin. The IncHI2/P multi replicon plasmid harboring *bla*_CTX-M-2_ was not confirmed as a multi replicon by Southern blot hybridization. However, similar IncHI2/P plasmids harboring *bla*_CTX-M-2_ were reported previously and have been confirmed as multi replicons [49]. Additional experiments such as PFGE, RFLP or sequence analysis of the whole plasmid are required to determine whether the plasmids with similar replicon types, pMLST-type or RST-type are clonally related. Based on the data presented in this study we cannot determine whether the overlapping genes and plasmids reside in *E. coli* that are host specific (animal or human) or that they proliferate well in both human and animal hosts.

In six herds, only promoter mutations of the chromosomal *ampC* gene were detected (Table 2) and no isolates harbouring plasmid mediated ESBL/AmpC genes were found within the same herd. Since this study focused on the dissemination of plasmid mediated ESBL/AmpC genes, this prevalence is likely to be an underestimate of the actual number of herds that are positive for chromosomal *ampC* genes with promoter variations that lead to non-wild type susceptibility to cefotaxime. Furthermore, we based the absence of plasmid mediated ESBL/AmpC genes on array data. Novel genes or ESBL/AmpC variants not present on the array will have been missed. The phenotypic combination disk test was performed on all isolates negative in the array in order to reduce the chance of missing novel genes.

The fact that international trafficking of live calves from many different dairy farms to Dutch veal calf farms happens on a large scale may contribute to the high dissemination observed in this study. This may also explain the high diversity in gene/plasmid combinations compared to Dutch poultry, in which *bla*_CTX-M-1_ is highly prevalent and commonly associated with IncI1 [36].

### Conclusion

A relatively high percentage of slaughter batches (66%) were found positive for *E. coli* with a non-wild type susceptibility to cefotaxime. The within-herd prevalence varied greatly from zero to 90% positive. In the majority of herds positive for ESBL/AmpC-producing *E. coli*, the within-farm prevalence was below 50%. Furthermore, plasmid mediated resistance to cefotaxime was predominantly caused by enzymes encoded by the *bla*_CTX-M_ gene family. Many gene-plasmid combinations found in this study have also been found in cattle in other countries, indicating that there is a non-local dissemination of resistance determinants. However, since the number of ESBL/AmpC-producing *E. coli* was not quantified, no conclusions can be drawn about the actual risk of presence.
